# In utero Zika virus exposure and neurodevelopment at 24 months in toddlers normocephalic at birth: a cohort study

**DOI:** 10.1186/s12916-020-01888-0

**Published:** 2021-01-21

**Authors:** Rebecca Grant, Olivier Fléchelles, Benoît Tressières, Mama Dialo, Narcisse Elenga, Nicolas Mediamolle, Adeline Mallard, Jean-Christophe Hebert, Noémie Lachaume, Elvire Couchy, Bruno Hoen, Arnaud Fontanet

**Affiliations:** 1 Emerging Diseases Epidemiology Unit, Institut Pasteur, Paris, France; 2 Sorbonne Université, Paris, France; 3 CHU de la Martinique, Fort-de-France, Martinique France; 4 Centre d’Investigation Clinique Antilles – Guyane, Pointe-à-Pitre, France; 5 CHU de la Guadeloupe, Pointe-à-Pitre, France; 6 CH Cayenne, Cayenne, French Guiana; 7 CH Basse-Terre, Basse-Terre, France; 8 Conservatoire National des Arts et Métiers, Paris, France

**Keywords:** Zika virus, Emerging infectious diseases, Neurodevelopment, Pediatrics, Epidemiology

## Abstract

**Background:**

In utero exposure to Zika virus (ZIKV) is known to be associated with birth defects. The impact of in utero ZIKV exposure on neurodevelopmental outcomes in early childhood remains unclear. The objective of this study was to determine the impact of in utero ZIKV exposure on neurodevelopment at 24 months of age among toddlers who were born normocephalic to women who were pregnant during the 2016 ZIKV outbreak in French territories in the Americas.

**Methods:**

We conducted a population-based mother-child cohort study of women whose pregnancies overlapped with the 2016 ZIKV epidemic in Guadeloupe, Martinique, and French Guiana. Infants were included in this analysis if maternal ZIKV infection during pregnancy could be determined, the newborn had a gestational age ≥ 35 weeks, there were no abnormal transfontanelle cerebral ultrasound findings after delivery or no abnormal ultrasound findings on the last ultrasound performed during the third trimester of the mother’s pregnancy, there was an absence of microcephaly at birth, and the parent completed the 24-month neurodevelopment assessment of the infant at 24 months (± 1 month) of age. ZIKV exposure of the toddler was determined by evidence of maternal ZIKV infection during pregnancy. Neurodevelopment assessments included the Ages and Stages Questionnaire (ASQ) for five dimensions of general development—communication, gross motor, fine motor, problem solving, and personal-social skills; the Modified Checklist for Autism on Toddlers (M-CHAT) for behavior; and the French MacArthur Inventory Scales (IFDC) for French language acquisition.

**Results:**

Between June 2018 and August 2019, 156 toddlers with and 79 toddlers without in utero ZIKV exposure completed neurodevelopment assessments. Twenty-four (15.4%) ZIKV-exposed toddlers and 20 (25.3%) ZIKV-unexposed toddlers had an ASQ result below the reference − 2SD cut-off (*P* = 0.10) for at least one of the five ASQ dimensions.

**Conclusion:**

In one of the largest population-based cohorts of in utero ZIKV-exposed, normocephalic newborns to date, there were minimal differences apparent in neurodevelopment outcomes at 24 months of age compared to ZIKV-unexposed toddlers at 24 months of age.

**Trial registration:**

ClinicalTrials.gov, NCT02810210. Registered 20 June 2016.

## Background

Zika virus (ZIKV) infection during pregnancy is understood to cause neurological complications in the fetus, collectively known in its most severe form as congenital Zika syndrome (CZS) [1]. The 2015–2016 ZIKV epidemic enabled the risk of adverse pregnancy outcomes associated with ZIKV infection during pregnancy to be estimated [2–5]. However, adverse pregnancy outcomes and CZS do not cover the full spectrum associated with in utero ZIKV exposure, and the characterization and risk of developmental outcomes beyond birth remain unclear [6]. The newborns who were born to women whose pregnancy occurred during the ZIKV epidemic are now in early childhood and the development of these young children needs to be assessed, as well as the risk for abnormal development attributable to in utero ZIKV exposure.

Adverse neurodevelopment findings in infants exposed in utero to ZIKV with and without CZS have recently been documented in the USA [7], Brazil [8–11], and Colombia [12]. Yet, within these prospective studies assessing neurodevelopment outcomes, there has been minimal use of comparative and appropriate control groups. This is a critical consideration, particularly given that neurodevelopment in early childhood is known to be influenced by both genetic and environmental factors, including substance abuse during pregnancy, maternal age [13], sociodemographic determinants [14], prematurity or low birth weight, maternal and early infant nutritional status [15], and sex of the infant in the case of language acquisition [13].

To study the impact of in utero ZIKV exposure on pregnancy and early child development outcomes, the ZIKA-DFA-FE (pregnant women) and ZIKA-DFA-BB (newborns) prospective cohort studies were established in Guadeloupe, Martinique, and French Guiana in 2016. The dynamics of the 2016 ZIKV epidemic in the three French territories were such that they enable the ZIKA-DFA-BB cohort study to closely examine the impact of in utero ZIKV exposure on early child outcomes beyond birth. Here, we present the neurodevelopment assessment results at 24 months of age. The objective of the study was to compare the neurodevelopment outcomes to toddlers who were exposed to ZIKV in utero with those who were not exposed to estimate the impact of in utero ZIKV exposure on neurodevelopmental outcomes at 24 months.

## Methods

All women whose pregnancy overlapped with the 2016 ZIKV epidemic in Guadeloupe, Martinique, or French Guiana who sought antenatal care at one of the participating hospitals were invited to participate in the ZIKA-DFA-FE prospective cohort study of pregnant women, for which the recruitment and ZIKV testing strategies have been previously described [3].

ZIKA-DFA-BB was a prospective cohort study of toddlers born to women whose pregnancies overlapped with the 2016 ZIKV epidemic in French territories in the Americas: Guadeloupe, Martinique, and French Guiana. Toddlers were followed up in routine pediatric consultation until 24 months of age.

### Inclusion in analysis

Toddlers were included in this analysis if all of the following criteria were met: maternal ZIKV infection during pregnancy could be determined; the newborn had a gestational age of 35 weeks or more; there were no abnormal transfontanelle cerebral ultrasound findings after delivery up to 2 months of age or, in the absence of a transfontanelle cerebral ultrasound after delivery, no abnormal ultrasound findings on the last ultrasound performed during the third trimester of the mother’s pregnancy; there was an absence of microcephaly at birth, defined as a head circumference above − 2SD below the mean according to sex and gestational age on the INTERGROWTH-21st chart; and the parent completed the neurodevelopment assessment of the toddler at 24 months (± 1 month) of age.

### Exposure assessment

In utero ZIKV exposure was determined by RT-PCR or serological evidence of ZIKV infection in serum and/or urine samples from the mother collected during pregnancy. A toddler was considered exposed to ZIKV if the mother had RT-PCR (RealStar Zika Virus RT-PCR Kit 1.0, Altona Diagnostics) positive result for ZIKV in blood, urine, or both at any stage during her pregnancy; if the toddler had anti-ZIKV IgM (EuroImmun ELISA or in-house MAC-ELISA in French Guiana [16]) in the cord blood or blood taken within the first 10 days of life; or if the toddler had anti-ZIKV IgG (EuroImmun ELISA or in-house MAC-ELISA in French Guiana [16]) in the blood beyond 12 months of age and the date of birth was posterior to the end of the ZIKV epidemic: 11 September 2016 in French Guiana, 25 September 2016 in Guadeloupe, and 16 October 2016 in Martinique.

Anti-ZIKV kinetic studies have shown that anti-ZIKV IgG antibodies appear rapidly after infection and remain detectable up to 6 months with 100% sensitivity [17–19]. Therefore, a negative serology for anti-ZIKV IgG in mothers at the time of delivery has a 100% negative predictive value for ZIKV infection in the mother during pregnancy and was used to define toddlers as unexposed to ZIKV in utero*.*

### Neurodevelopment assessment

Three pediatric neurodevelopment evaluation tools were used to assess neurodevelopment and administered to a parent/legal guardian at the time of the 24-month pediatric consultation of the toddler. All questionnaires were administered in French.

The 30-item parent-reported screening test Ages and Stages Questionnaire-III (ASQ), previously validated in France [20], was used to identify toddlers at risk for developmental delay across five dimensions: communication, gross motor, fine motor, problem solving, and personal-social skills [21]. Each dimension consists of six questions, with possible responses: “yes” (10 points) if the child performs the behavior, “sometimes” (5 points), and “not yet” (0 points). A score for each dimension was calculated by adding the points from each dimension and each dimensional score reflected the child’s ability within that dimension. Abnormal ASQ outcomes were described as a dimension score below validated cut-off values, set at 2SD below the mean using reference norms [21].

The 23-item parent-reported screening test Modified Checklist for Autism on Toddlers (M-CHAT) was used to identify toddlers at risk for behavior disorder [22]. A total score was calculated by adding the points from the screening test, with low risk defined as a total score of 2 or lower, medium risk 3–7 points, and high risk 8 points or above. A positive M-CHAT screen was considered as having a total score of 3 or above.

The French MacArthur-Bates communicative development inventories (Inventaires français du développement communicatif (IFDC)) was used to assess French language acquisition for which the parent identified from a validated list of 100 words the words that the toddler says spontaneously [23]. Total word count was calculated as the sum of the words the toddler says spontaneously. Abnormal IDFC outcomes were described as total word count below validated 10th percentile thresholds derived from a reference population [23].

### Statistical analysis

The maternal and newborn characteristics of the in utero ZIKV-exposed and ZIKV-unexposed toddlers were compared using Student’s *t* test for continuous variables and chi-squared test for categorical variables. Missing values for questions within each ASQ dimension were replaced by the average of the other questions in that same dimension.

Univariable and multivariable logistic regression models were used to evaluate the association between in utero ZIKV exposure and abnormal ASQ score per dimension.

For all statistical analyses, *P* < 0.05 was considered statistically significant. All statistical analyses were performed using R, version 3.6.1.

## Results

**Fig. 1:**
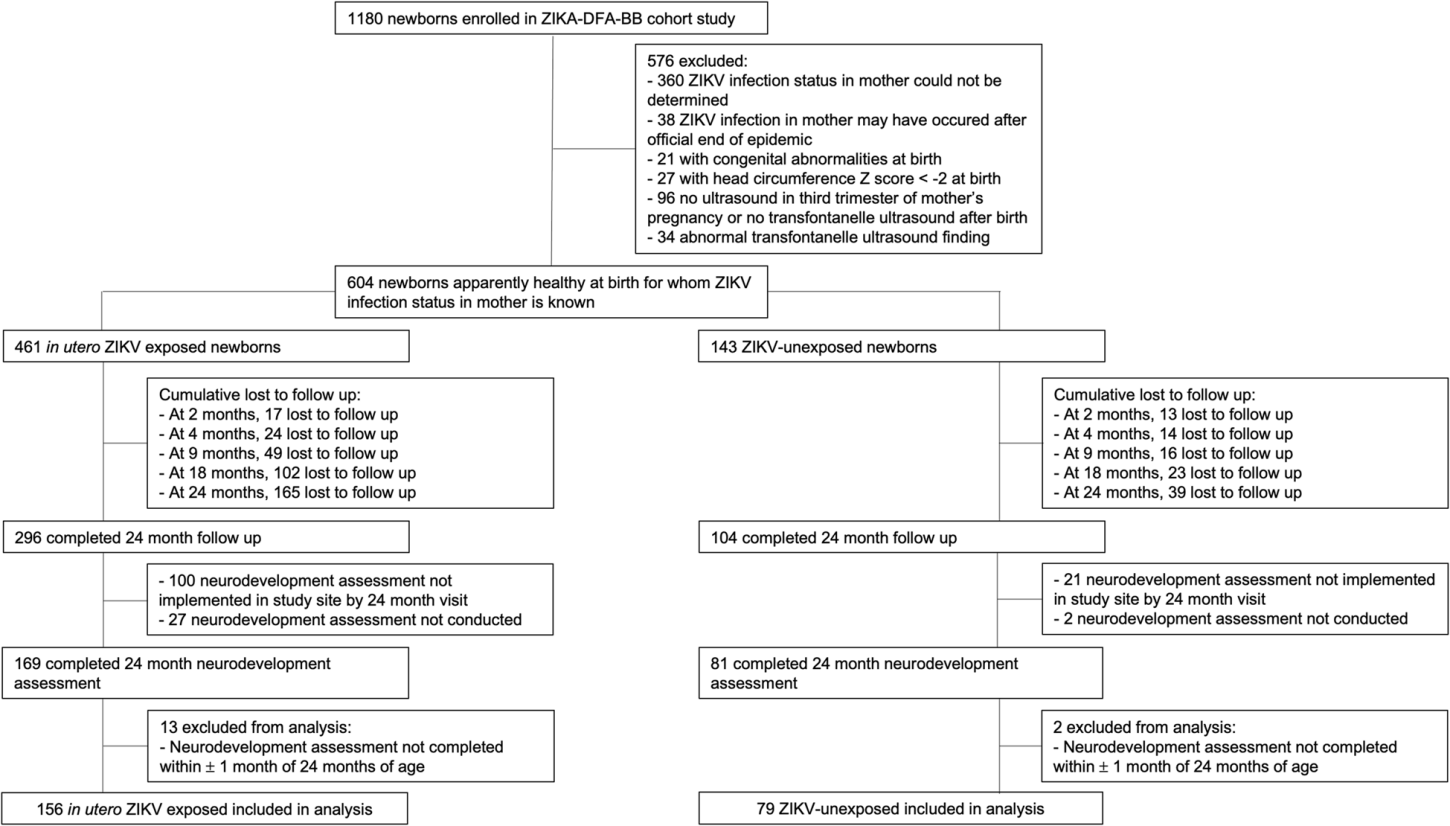
Toddlers from the ZIKA-DFA-BB prospective cohort study in Guadeloupe, Martinique, and French Guiana included in the analysis

From June 2018 to August 2019, 1180 newborns were enrolled in the ZIKA-DFA-BB cohort study. Of these, 572 were excluded from analysis: ZIKV infection status of the mother could not be determined (360 newborns), or it could not be excluded that maternal ZIKV infection preceded the start of the pregnancy (38 newborns), or the newborn had congenital abnormalities at birth (21 newborns), or the newborn had a head circumference *Z* score of − 2SD or lower at birth (27 infants), or the newborn had no ultrasound findings available from the third trimester of the mother’s pregnancy (96 newborns), or the newborn had an abnormal transfontanelle cerebral ultrasound findings at birth up to 2 months of age (34 newborns). Of the remaining 604 newborns, 247 completed the 24-month neurodevelopment assessment (169 toddlers born to women with confirmed ZIKV infection during pregnancy, 81 toddlers born to women with no ZIKV infection during pregnancy). A further 15 toddlers were excluded from the analysis as the neurodevelopment assessment was not administered within ± 1 month of the toddlers 24 months of age. We were therefore able to describe the results of the neurodevelopment assessment at 24 months for 235 toddlers: 156 in utero ZIKV-exposed toddlers and 79 ZIKV-unexposed toddlers (Fig. 1).

Table 1 shows the maternal and newborn characteristics of the 235 toddlers by ZIKV exposure status. Of those included in the analysis, no abnormalities associated with ZIKV exposure were reported in routine pediatric consultation at birth. Additional file 1: Supplementary Table 1 shows the RT-PCR and serological evidence of maternal ZIKV infection. Comparisons between ZIKV-exposed and ZIKV-unexposed toddlers indicated a lower maternal age (*P =* 0.01), higher maternal education (*P* = 0.04), and higher paternal education (*P* = 0.04) in the ZIKV unexposed; a higher proportion of toddlers from Guadeloupe in the ZIKV-exposed group and a higher proportion of toddlers from Martinique in the ZIKV-unexposed group (*P* ≤ 0.001); higher parity in the ZIKV exposed (*P* = 0.04); and greater use of mosquito repellents in the ZIKV-exposed group (*P* = 0.05).

**Table 1:** Maternal and newborn characteristics of 235 toddlers included in the analysis by ZIKV exposure status

	In utero ZIKV exposure (*N* = 156)	ZIKV unexposed (*N* = 79)	*P* value
Maternal characteristics			
Age at time of pregnancy (years)			
Mean ± SD	30.7 ± 6.3	28.6 ± 6.0	*0.01*
Interquartile range	26–36	25–33	
Occupation—*n* (%)			*< 0.001*
- Student	5 (3.2)	3 (3.8)	
- Self-employed/business owner/farmer	11 (7.1)	2 (2.5)	
- Executive/highly skilled worker	22 (14.1)	10 (12.7)	
- Intermittent profession	10 (6.4)	24 (30.4)	
- Salaried employee	60 (38.5)	16 (20.2)	
- Not employed	47 (30.1)	24 (30.4)	
- Unknown or declined to respond	1 (0.6)	0 (0)	
Educational attainment of mother—*n* (%)			*0.04*
- Primary	30 (19.2)	7 (8.9)	
- Secondary	47 (30.1)	31 (39.2)	
- Tertiary	66 (42.3)	39 (49.4)	
- Unknown or declined to respond	13 (8.3)	2 (2.5)	
Educational attainment of father—*n* (%)			*0.04*
- Primary	22 (14.1)	4 (5.1)	
- Secondary	39 (25.0)	28 (35.4)	
- Tertiary	32 (20.5)	22 (27.8)	
- Unknown or declined to respond	62 (39.7)	25 (31.6)	
Residence—*n* (%)			*< 0.001*
- Guadeloupe	88 (56.4)	12 (15.1)	
- Martinique	65 (41.7)	63 (79.7)	
- French Guiana	3 (1.9)	4 (5.1)	
Parity—*n* (%)			*0.05*
0	64 (41.0)	41 (51.9)	
1	51 (32.7)	27 (34.2)	
2	25 (16.0)	3 (3.9)	
3+	16 (10.3)	8 (10.1)	
Previous adverse pregnancy outcomes—*n* (%)			
- Congenital abnormalities	0 (0)	0 (0)	–
- Stillbirth*	6 (3.8)	0 (0)	0.18
- Medical termination of pregnancy*	1 (0.6)	0 (0)	1
Lifestyle practices during 2016–2017 pregnancy—*n* (%)			
- Alcohol consumption*	1 (0.6)	0 (0)	1
- Drug use*	1 (0.6)	1 (1.4)	1
- Smoking*	5 (3.2)	5 (6.8)	0.38
- Use of mosquito repellents*	116 (75.3)	47 (61.0)	*0.04*
- Use of larvicides*	91 (59.5)	44 (59.5)	1
Newborn characteristics			
Gestational age (weeks)			
Mean ± SD	39.1 ± 1.3	39.1 ± 1.5	0.84
Delivery type—*n* (%)*			
Cesarean	29 (18.6)	8 (10.1)	0.13
- Guadeloupe	17/88 (19.3)	2/12 (16.7)	1
- Martinique	10/64 (15.6)	5/63 (7.9)	0.29
- French Guiana	2/3 (66.7)	1/4 (25.0)	0.49**
Sex—*n* (%)			
Male	70 (44.9)	40 (50.6)	0.49
Birth weight (g)			
Mean	3176 ± 476	3196 ± 451	0.75
Medical consultations since birth—*n* (%)*			
- Hospitalization > 1 day	33 (21.1)	17 (21.5)	1
- Emergency room consultation	84 (54.5)	52 (65.8)	0.13
- Osteopath consultation	28 (18.1)	13 (16.7)	0.93
- Psychomotor therapist consultation	3 (2.0)	1 (1.3)	1
- Psychologist consultation	1 (0.6)	0 (0)	1
- Speech therapist consultation	5 (3.2)	0 (0)	0.26
- Chest physiotherapist consultation	44 (28.2)	29 (35.4)	0.32
- Physiotherapist consultation	6 (3.9)	2 (2.6)	0.89

*Data are missing for stillbirth = 1; medical termination of pregnancy = 1; alcohol consumption = 6; drug use = 6; smoking = 6; mosquito repellent use = 4; larvicide use = 8; delivery type = 1; emergency room consultation = 2; osteopath consultation = 2; psychomotor consultation = 3; psychologist consultation = 1; speech therapist consultation = 2; physiotherapist consultation = 2

**Fisher’s exact test

Comparisons between the toddlers included in the analysis (*N* = 235) and those who were excluded because they did not complete the neurodevelopment assessment (*N* = 369) revealed higher completion of the questionnaire in Martinique and lower completion of the questionnaire in French Guiana in exposed (*P* ≤ 0.001) and unexposed groups (*P* ≤ 0.001); higher completion rate of the questionnaire in toddlers born to women with lower parity in the unexposed groups (*P* ≤ 0.001); and higher completion rate in toddlers born to women who did not consume alcohol during pregnancy (*P* = 0.04) (data not shown).

Table 2 shows the mean ASQ results per dimension by ZIKV exposure status, as well as the number of toddlers in each group with a score below the − 2SD cut-off. The communication dimension showed a statistically significant difference between the two groups, with the ZIKV exposed having a higher mean communication score (49.5 vs 44.0; *P* = 0.01) and a smaller proportion of toddlers falling below the − 2SD cut-off value (8.3% vs 20.3%; *P* = 0.02). The dimensions most frequently scoring below the threshold in the ZIKV-exposed and ZIKV-unexposed groups were communication and personal-social skills.

**Table 2:** ASQ results of 235 toddlers included in the analysis by ZIKV exposure status

	In utero ZIKV exposure (*N* = 156)		ZIKV unexposed (*N* = 79)		Comparison of means	Comparison of *n* below the cut-off
	Mean (± SD)	*n* below − 2SD cut-off (%)	Mean (± SD)	*n* below − 2SD cut-off (%)	*P* value	*P* value
Communication	49.5 ± 12.6	13 (8.3)	44.0 ± 16.0	16 (20.3)	*0.01*	*0.02*
Gross motor	55.9 ± 7.6	5 (3.2)	53.4 ± 8.6	3 (3.8)	*0.04*	1
Fine motor	52.7 ± 9.0	4 (2.6)	52.7 ± 8.5	4 (5.1)	0.96	0.54
Problem solving	48.0 ± 12.2	8 (5.1)	47.4 ± 10.2	2 (2.5)	0.68	0.56
Personal-social	48.6 ± 9.8	11 (7.1)	46.7 ± 10.1	8 (10.1)	0.16	0.57

Additional file 1: Supplementary Table 2 shows the number of ASQ dimensions below the − 2SD cut-off value by ZIKV exposure. Twenty-four (15.4%) ZIKV-exposed toddlers and 20 (25.3%) ZIKV-unexposed toddlers had an ASQ result for at least one of the five ASQ dimensions below the reference − 2SD cut-off (*P* = 0.10).

Further analyses among the ZIKV-exposed toddlers demonstrated no difference in the proportion of toddlers with at least one ASQ dimension below the − 2SD cut-off value by trimester of pregnancy during which ZIKV infection occurred for the women who had ZIKV infection determined by RT-PCR (*P* = 0.67).

In univariable logistic regression analyses shown in Table 3, in utero ZIKV exposure and sex of the toddler were associated with an ASQ score for communication below the − 2SD cut-off. In multivariable regression analysis, shown in Table 4, in utero ZIKV exposure and sex of the toddler were identified as independent predictors of an ASQ score for communication below the − 2SD cut-off.

**Table 3:** Univariable logistic regression model predicting ASQ dimension result below the − 2SD cut-off for 235 toddlers included in the analysis by ZIKV exposure status

	OR (95% CI) of ASQ dimension below − 2SD cut-off									
Characteristic	Communication	*P* value	Gross motor	*P* value	Fine motor	*P* value	Problem solving	*P* value	Personal-social	*P* value
ZIKV exposure										
Exposed	0.36 (0.2–0.8)	*0.01*	0.84 (0.2–3.6)	0.81	0.49 (0.1–2.0)	0.32	2.08 (0.4–10.0)	0.36	0.67 (0.3–1.8)	0.41
Unexposed	1		1		1		1		1	
Sex										
Male	1		1		1		1		1	
Female	0.35 (0.2–0.8)	*0.01*	1.49 (0.4–6.4)	0.59	0.88 (0.2–3.6)	0.85	0.36 (0.1–1.4)	0.15	0.62 (0.2–1.6)	0.31
Education of mother										
Primary	1	0.57	1	0.65	1	0.65	1	0.58	1	0.15
Secondary	0.76 (0.3–2.3)		0.70 (0.1–4.4)		0.70 (0.1–4.4)		0.30 (0.1–1.9)		1.20 (0.2–6.5)	
Tertiary	0.54 (0.2–1.6)		0.34 (0.1–2.5)		0.34 (0.1–2.5)		0.45 (0.1–2.1)		1.44 (0.3–7.1)	
Unknown	1.29 (0.3–6.0)		1.25 (0.1–14.9)		1.25 (0.1–14.9)		0.81 (0.08–8.5)		6.36 (1.0–39.6)	
Education of father										
Primary	1	0.28	1	0.55	1	0.65	1	0.13	1	0.12
Secondary	0.52 (0.2–1.6)		0.56 (0.1–3.6)		0.70 (0.1–4.4)		0.56 (0.09–3.6)		0.49 (0.14–1.7)	
Tertiary	0.27 (0.07–1.1)		0.23 (0.02–2.6)		0.34 (0.05–2.5)		0 (0)		0.25 (0.05–1.1)	
Unknown	0.43 (0.1–1.3)		0.28 (0.04–2.1)		1.25 (0.1–14.9)		0.73 (0.1–4.0)		0.20 (0.05–0.8)	
Languages spoken in the household										
One	1		1		1		1		1	
More than one	2.00 (0.8–4.9)	0.13	0.98 (0.2–4.2)	0.98	0.58 (0.1–2.4)	0.44	2.43 (0.5–11.7)	0.27	1.00 (0.4–2.7)	0.99
Parity										
0	1	0.08	1	0.75	1	0.44	1	0.31	1	0.89
1	0.65 (0.2–1.8)		0.89 (0.2–5.5)		0.53 (0.1–2.8)		0.26 (0.03–2.3)		1.01 (0.3–3.0)	
2	1.29 (0.4–4.4)		2.62 (0.4–16.5)		0 (0)		1.54 (0.3–8.4)		0.93 (0.2–4.7)	
3+	3.19 (1.1–9.3)		1.48 (0.2–14.9)		0.87 (0.1–7.8)		1.82 (0.3–10.0)		1.73 (0.4–7.1)	
Birth weight										
Low birth weight	1.02 (0.2–4.7)	0.98	9.88 (2.1–45.9)	*0.003*	2.02 (0.2–17.5)	0.52	0		1.70 (0.4–8.1)	0.51
Normal	1		1		1		1		1

**Table 4:** Multivariable logistic regression model predicting ASQ dimension result below the − 2SD cut-off for 235 toddlers included in the analysis by ZIKV exposure status

	Adjusted OR (95% CI) of ASQ dimension below − 2SD cut-off									
Characteristic	Communication	*P* value	Gross motor	*P* value	Fine motor	*P* value	Problem solving	*P* value	Personal-social	*P* value
ZIKV exposure										
Exposed	0.26 (0.1–0.6)	*0.004*	0.71 (0.1–4.0)	0.70	0.47 (0.1–2.2)	0.33	1.73 (0.3–9.9)	0.54	0.63 (0.2–1.8)	0.40
Unexposed	1		1		1		1		1	
Sex										
Male	1		1		1		1		1	
Female	0.35 (0.1–0.9)	*0.03*	1.73 (0.3–8.8)	0.51	0.83 (0.2–3.7)	0.81	0.34 (0.1–1.5)	0.16	0.60 (0.2–1.7)	0.33
Education of mother										
Primary	1	0.71	1	0.87	1	0.64	1	0.52	1	0.10
Secondary	0.68 (0.2–2.5)		0.82 (0.1–7.3)		0.34 (0.04–2.8)		0.28 (0.04–2.1)		1.16 (0.2–7.3)	
Tertiary	0.57 (0.1–2.3)		0.39 (0.03–5.0)		0.32 (0.03–2.9)		0.81 (0.1–5.4)		1.53 (0.3–9.5)	
Unknown	1.65 (0.3–9.4)		1.22 (0.1–25.8)		1.37 (0.1–19.7)		1.05 (0.1–12.6)		9.54 (1.3–69.6)	
Education of father										
Primary	1	0.55	1	0.75	1	0.36	1	0.17	1	0.11
Secondary	0.47 (0.1–2.2)		1.35 (0.1–15.9)		1.05 (0.1–15.2)		0.66 (0.1–6.1)		0.57 (0.1–2.7)	
Tertiary	0.33 (0.1–1.9)		0.57 (0.02–15.7)		0 (0)		0 (0)		0.24 (0.04–1.6)	
Unknown	0.40 (0.1–1.5)		0.51 (0.05–4.9)		1.11 (0.1–13.4)		0.8 (0.1–5.6)		0.18 (0.04–0.9)	
Languages spoken in the household										
One	1		1		1		1	0.29	1	0.99
More than one	2.15 (0.8–5.7)	0.12	0.64 (0.13–3.2)	0.58	0.39 (0.1–1.8)	0.23	2.4 (0.4–12.9)		1.01 (0.4–2.9)	
Parity										
0	1	0.10	1	0.85	1	0.33	1	0.27	1	0.94
1	0.59 (0.2–1.8)		0.95 (0.1–6.8)		0.39 (0.1–2.3)		0.19 (0.02–1.8)		0.92 (0.3–3.0)	
2	1.15 (0.3–5.0)		2.25 (0.2–22.0)		0 (0)		0.77 (0.1–6.3)		0.58 (0.1–4.3)	
3+	3.22 (1.0–11.0)		0.67 (0.03–14.1)		0.43 (0.1–6.1)		1.60 (0.3–10.5)		1.14 (0.2–6.0)	
Birth weight										
Low birth weight	0.35 (0.1–2.5)	0.30	10.21 (1.7–61.3)	*0.01*	2.3 (0.2–30.1)	0.52	0 (0)		0.82 (0.1–6.4)	0.85
Normal	1		1		1		1		1	

Additional file 1: Supplementary Table 3 shows the M-CHAT behavior disorder risk by ZIKV exposure status. There was no difference observed in behavior disorder screening risk between ZIKV-exposed and ZIKV-unexposed toddlers (*P* = 0.15).

Additional file 1: Supplementary Table 4 shows language acquisition of 233 francophone toddlers by ZIKV exposure status. There were no observed differences in mean language acquisition (*P* = 0.36), nor in the proportion of toddlers below the 10th percentile threshold (*P* = 0.53). Further stratification by sex and by the number of languages spoken in the household did not identify any differences by in utero ZIKV exposure status.

## Discussion

In one of the largest population-based, mother-child cohorts of in utero ZIKV-exposed normocephalic at birth to date, we found no apparent differences in neurodevelopment outcomes compared to ZIKV-unexposed toddlers at 24 months of age.

We found 24 (15.3%) toddlers with in utero ZIKV exposure at risk of neurodevelopment delay using the ASQ neurodevelopment screening tool. This is largely comparable to neurodevelopmental findings in other prospective ZIKV cohort studies published to date. Lopes Moreira et al. [8] used the Bayley Scales of Infant and Toddler Development-III to assess 94 children who had also undergone neuroimaging between 12 and 18 months of age. Twenty-four (25.5%) toddlers were found to have at least one abnormal neurodevelopment finding. Considering only the 59 with normal imaging for comparison with our findings, the number with abnormal neurodevelopment was 13 (13.8%). Einspieler et al. [10] followed 56 toddlers without microcephaly at birth and who were born to women with RT-PCR positive result for ZIKV during pregnancy. Ten (17.9%) toddlers were found to have an adverse neurologic exam or neurodevelopment assessment at 12 months of age.

Among in utero ZIKV-exposed infants, irrespective of abnormalities at birth, Rice et al. [7] assessed follow-up care reports of 1450 toddlers with in utero ZIKV exposure with and without ZIKV-associated birth defects aged 1 year of age or older and found 9% had a least one neurodevelopment abnormality possibly associated with in utero ZIKV exposure. Nielsen-Saines et al. [9] used the Bayley Scales of Infant and Toddler Development-III at 18 months of age and described the neurodevelopment outcomes of 146 toddlers born to women with rash and RT-PCR ZIKV infection during pregnancy. Forty-one (28.1%) toddlers were found to have abnormal neurodevelopment, with language having the greatest proportion of toddlers with an abnormal outcome. In this respect, we have limited our analyses to those toddlers who were normocephalic at birth, defined by an absence of microcephaly at birth, and by an absence of abnormal transfontanelle cerebral findings after delivery or of ultrasound findings during the third trimester of the mother’s pregnancy. The results of assessments of the toddlers excluded from our analysis with CZS or abnormal ultrasound findings will be described in future publications.

The important contribution of our study is the comparison of neurodevelopment outcomes of toddlers with in utero ZIKV exposure with toddlers who were known to have no in utero exposure to ZIKV*.* Of the 79 toddlers born to women with no evidence of ZIKV infection at the time of delivery, we found 20 (25.3%) to have an abnormal neurodevelopment finding using the ASQ neurodevelopment assessment tool. Further, there were no differences between ZIKV-exposed and ZIKV-unexposed toddlers for behavior disorder screening risk (*P* = 0.15), nor for language acquisition, whether by mean (*P* = 0.36) or the proportion below the 10th percentile threshold (*P* = 0.53). These findings underscore the pertinence of a comparative control group for determining the risk of adverse outcomes—whether during pregnancy or into early childhood—which may be attributable to ZIKV infection. Previous findings of prospective ZIKV cohort studies have attributed abnormal neurodevelopment in early childhood solely to in utero ZIKV exposure. Our findings would suggest that this attribution may not necessarily be warranted and that other factors which are known to be associated with neurodevelopment in early childhood need to be accounted for when determining these risk estimates.

While we found communication and personal-social skills to be those most frequently below the − 2SD threshold in the ZIKV exposed and the ZIKV unexposed, we also found in univariable and multivariable logistic regression analyses that in utero ZIKV exposure was an independently protective factor against an abnormal ASQ dimension score for communication. The strength of this association is such that the likelihood that this is a spurious finding is minimal. Further, the multivariable logistic regression analysis shown in Table 4 indicates that the in utero ZIKV exposure protective effect is not confounded. Recall bias is unlikely, as the statistically significant difference between ZIKV exposed and ZIKV unexposed was only observed for communication and not across the other ASQ dimensions, as would perhaps be expected if the parent/legal guardian—aware of the ZIKV exposure status of the toddler—responded more positively to the questionnaire in the in utero ZIKV-exposed group compared to the ZIKV-unexposed group. Our finding is further strengthened by the fact that the multivariable logistic regression model identified other risk factors for neurodevelopment in early childhood that have been previously identified, including parity and sex of the toddler [13, 24]. This suggests that the ASQ screening tool itself is an appropriate tool in this setting, that it has performed well in identifying risk factors for those at risk of abnormal neurodevelopment, and that the communication finding may be influenced by a factor which has not been quantified as part of our analyses, such as parent stimulation in early childhood, or eagerness from the parents of ZIKV-exposed toddlers to emphasize attainment of developmental milestones, more so than perhaps the parents of the ZIKV-unexposed toddlers.

The results of our study are strengthened by the study design. In addition to the inclusion of a comparative control group, as described above, the ZIKA-DFA-FE and ZIKA-DFA-BB cohort studies were conducted in an unselected population-based cohort, in that both the exposed and unexposed groups were derived from the same source population. While we cannot rule out that self-selection in the cohort study may have occurred, the external validity of the findings is nonetheless strengthened by the study design. The prospective nature of the ZIKA-DFA-FE pregnancy cohort, followed by the ZIKA-DFA-BB pediatric cohort, enabled us to capture acute ZIKV infection during pregnancy—the majority by RT-PCR—and then to follow development outcomes into early childhood.

One limitation of our study is the loss to follow-up of toddlers from delivery to 24 months of age. However, maintaining toddlers in an extended follow-up has proved challenging for almost all ZIKV pediatric cohorts to date [7, 9]. Healthy toddlers are generally more difficult to maintain in an extended follow-up cohort study. At this stage, we cannot exclude a selection bias that may have resulted in toddlers who were lost to follow-up in the control group had normal neurodevelopment and their loss to follow-up may have artificially inflated the proportion of toddlers in the control group with abnormal neurodevelopment findings. However, the comparison of medical consultations outside routine pediatric consultations did not differ between the ZIKV exposed and the ZIKV unexposed, as would be expected if the control group was somehow different in terms of non-ZIKV-related disease status compared to the ZIKV-exposed group. Further, one of the principal reasons for the loss to follow-up and non-completion of the neurodevelopment assessment was the unavailability of the neurodevelopment assessment in each of the study sites by the time of the 24-month visit. Finally, we used developmental screening tools, rather than neurodevelopment and/or behavior evaluation diagnostic tools. The comparative ease-of-use of the screening tools and budget-related constraints related to the requirement of a trained psychologist or psychotherapist led to the decision to use the various screening tools. Nonetheless, the ASQ-III tool has been widely used in studies to assess the neurodevelopment of infants and toddlers [25–31].

## Conclusions

Overall, we found 15.3% of toddlers exposed to ZIKV in utero to have abnormal neurodevelopment findings at 24 months of age, a finding largely consistent with the results of other cohort studies published to date. However, when comparing this result with the result of toddlers not exposed to ZIKV in utero in our cohort study, we found no statistically significant difference. Therefore, in the absence of congenital abnormalities or abnormal ultrasound findings in the final stages of pregnancy or at delivery, there would not appear to be an impact on longer term neurodevelopment outcomes attributable to in utero ZIKV exposure which may manifest after birth up to 24 months of age. However, it is important that these neurodevelopment assessments are continued into early childhood.

## Supplementary Information

**Additional file 1: Supplementary Table 1**. RT-PCR and serological evidence of maternal ZIKV infection during pregnancy of 235 infants included in this analysis. **Supplementary Table 2**. ASQ dimensions below -2SD cut-off value of 235 infants included in this analysis by ZIKV exposure. **Supplementary Table 3**. M-CHAT behavior disorder risk of 235 infants included in analysis by ZIKV exposure status. **Supplementary Table 4**. IFDC language acquisition of 233 francophone infants included in analysis by ZIKV exposure status.

## Data Availability

The datasets used and/or analyzed during the current study are available from the corresponding author on reasonable request.

## Abbreviations

ASQ: Ages and Stages Questionnaire
CZS: Congenital Zika syndrome
IFDC: French MacArthur Inventory Scales
IgG: Immunoglobulin G
IgM: Immunoglobulin M
M-CHAT: Modified Checklist for Autism on Toddlers
RT-PCR: Reverse transcriptase polymerase chain reaction
ZIKV: Zika virus
