# Supplementary material for: In utero Zika virus exposure and neurodevelopment at 24 months in toddlers normocephalic at birth: a cohort study
Source: BMC Med. 2021 Jan 21;19:12. doi: 10.1186/s12916-020-01888-0 (PMC7819189; doi:10.1186/s12916-020-01888-0)
Supplement: Supplementary file 1 — Additional file 1: Supplementary Table 1. RT-PCR and serological evidence of maternal ZIKV infection during pregnancy of 235 infants included in this analysis. Supplementary Table 2. ASQ dimensions below -2SD cut-off value of 235 infants included in this analysis by ZIKV exposure. Supplementary Table 3. M-CHAT behavior disorder risk of 235 infants included in analysis by ZIKV exposure status. Supplementary Table 4. IFDC language acquisition of 233 francophone infants included in analysis by ZIKV exposure status. [file 12916_2020_1888_MOESM1_ESM.docx]

**Additional file 1: Supplementary Material**

***In utero* Zika virus exposure and neurodevelopment at 24 months in toddlers normocephalic at birth: A cohort study**

Additional file 1: Supplementary Table 1. RT-PCR and serological evidence of maternal ZIKV infection during pregnancy of 235 infants included in this analysis

Additional file 1: Supplementary Table 2. ASQ dimensions below -2SD cut-off value of 235 infants included in this analysis by ZIKV exposure

Additional file 1: Supplementary Table 3. M-CHAT behavior disorder risk of 235 infants included in analysis by ZIKV exposure status

Additional file 1: Supplementary Table 4. IFDC language acquisition of 233 francophone infants included in analysis by ZIKV exposure status

Additional file 1: Supplementary Table 1. RT-PCR and serological evidence of maternal ZIKV infection during pregnancy of 235 infants included in this analysis

|  | N |
| --- | --- |
| Symptomatic women with RT-PCR positive ZIKV infection during pregnancy |  |
| - ZIKV infection during first trimester | 46 |
| - ZIKV infection during second trimester | 51 |
| - ZIKV infection during third trimester | 16 |
| Asymptomatic women with RT-PCR positive ZIKV infection during pregnancy |  |
| - ZIKV infection during first trimester | 1 |
| - ZIKV infection during second trimester | 3 |
| - ZIKV infection during third trimester | 0 |
| Women with serological evidence of acute ZIKV infection during pregnancy | 42 |
| Women with no serological evidence of ZIKV infection at the end of pregnancy | 79 |

Additional file 1: Supplementary Table 2. ASQ dimensions below -2SD cut-off value of 235 infants included in analysis by ZIKV exposure status

| Number of ASQ dimensions below -2SD cut-off | *In utero* ZIKV exposure (N=156) | ZIKV unexposed (N=79) | P value |
| --- | --- | --- | --- |
|  |  |  | 0.22 |
| 0 | 132 (84.6) | 59 (74.7) |  |
| 1 | 15 (9.6) | 12 (15.1) |  |
| 2 | 5 (3.2) | 4 (5.1) |  |
| 3 | 2 (1.3) | 3 (3.8) |  |
| 4 | 0 (0) | 1 (1.3) |  |
| 5 | 2 (1.3) | 0 (0) |  |

Additional file 1: Supplementary Table 3. M-CHAT behavior disorder risk of 235 infants included in analysis by ZIKV exposure status

|  | *In utero* ZIKV exposure (N=156) | ZIKV unexposed (N=79) | P value |
| --- | --- | --- | --- |
|  |  |  | 0.15 |
| No risk | 118 (75.6) | 52 (65.8) |  |
| At risk | 38 (24.4) | 27 (34.1) |  |

Additional file 1: Supplementary Table 4. IFDC language acquisition of 233 francophone infants included in analysis by ZIKV exposure status

|  | *In utero* ZIKV exposure (N=154) | | | ZIKV unexposed (N=79) | | | Comparison of means | Comparison of n below cut-off |
| --- | --- | --- | --- | --- | --- | --- | --- | --- |
|  | N | Mean  (± SD) | n below 10^th^ percentile cut-off (%) | N | Mean  (± SD) | n below 10^th^ percentile cut-off (%) |  |  |
| Word count | 154 | 54.6 ± 32.0 | 45 (29.2) | 79 | 50.5 ± 33.3 | 27 (34.2) | 0.36 | 0.53 |
| Stratification |  |  |  |  |  |  |  |  |
| Sex |  |  |  |  |  |  |  |  |
| - Male | 69 | 51.7 ± 32.0 | 24 (34.8) | 41 | 44.3 ± 32.9 | 18 (45.0) | 0.25 | 0.39 |
| - Female | 85 | 57.0 ± 32.0 | 21 (24.7) | 39 | 56.9 ± 32.9 | 9 (23.1) | 0.98 | 1 |
| Languages spoken in household |  |  |  |  |  |  |  |  |
| - One | 54 | 60.5 ± 32.7 | 13 (24.1) | 31 | 52.0 ± 32.5 | 8 (25.8) | 0.25 | 1 |
| - More than one | 100 | 51.4 ± 31.3 | 32 (32.0) | 48 | 49.5 ± 34.1 | 19 (39.6) | 0.74 | 0.47 |
